# Exploring the Mechanism of Action of Herbal Medicine (*Gan-Mai-Da-Zao* Decoction) for Poststroke Depression Based on Network Pharmacology and Molecular Docking

**DOI:** 10.1155/2021/2126967

**Published:** 2021-08-21

**Authors:** Zhicong Ding, Fangfang Xu, Qidi Sun, Bin Li, Nengxing Liang, Junwei Chen, Shangzhen Yu

**Affiliations:** ^1^Jinan University, Guangzhou 510632, China; ^2^Yangzhou University, Yangzhou 225009, China; ^3^Wuyi Hospital of Traditional Chinese Medicine, Jiangmen 529000, China

## Abstract

**Background:**

Poststroke depression (PSD) is the most common and serious neuropsychiatric complication occurring after cerebrovascular accidents, seriously endangering human health while also imposing a heavy burden on society. Nevertheless, it is difficult to control disease progression. *Gan-Mai-Da-Zao* Decoction (GMDZD) is effective for PSD, but its mechanism of action in PSD is unknown. In this study, we explored the mechanism of action of GMDZD in PSD treatment using network pharmacology and molecular docking. *Material and methods*. We obtained the active components of all drugs and their targets from the public database TCMSP and published articles. Then, we collected PSD-related targets from the GeneCards and OMIM databases. Cytoscape 3.8.2 was applied to construct PPI and composite target disease networks. In parallel, the DAVID database was used to perform GO and KEGG enrichment analyses to determine the biological processes enriched in the treatment-related drugs in vivo. Finally, molecular docking was used to verify the association between the main active ingredients and their targets.

**Results:**

The network pharmacological analysis of GMDZD in PSD revealed 107 active ingredients with important biological effects, including quercetin, luteolin, kaempferol, naringenin, and isorhamnetin. In total, 203 potential targets for the treatment of this disease were screened, including STAT3, JUN, TNF, TPT53, AKT1, and EGFR. These drugs are widely enriched in a series of signaling pathways, such as TNF, HIF-1, and toll-like receptor. Moreover, molecular docking analysis showed that the core active components were tightly bound to their core targets, further confirming their anti-PSD effects.

**Conclusion:**

This prospective study was based on the integrated analysis of large data using network pharmacology technology to explore the feasibility of GMDZD for PSD treatment that was successfully validated by molecular docking. It reflects the multicomponent and multitarget characteristics of Chinese medicine and, more importantly, brings hope for the clinical treatment of PSD.

## 1. Introduction

Poststroke depression (PSD) is one of the most common and severe neuropsychiatric complications after stroke [1, 2]; it often starts insidiously, with mild symptoms of malaise and drowsiness in the early stages. Moreover, if patients with PSD are unable to express their feelings clearly due to language or cognitive impairment, the diagnosis is often compromised, and treatment is delayed [3]. This not only poses a great challenge to clinical work but also adds a heavy burden to the society and economy. A recent statistical study showed that approximately 795,000 people suffer from stroke each year in the United States. In detail, approximately 610,000 people experience stroke for the first time, and approximately 185,000 people experience recurrent stroke; more than 100,000 of these people die from stroke [4]. A meta-analysis of longitudinal studies showed that the prevalence of depression was 29% (95% CI 25–32) and remained stable up to 10 years after stroke, with a cumulative incidence of 39–52% within 5 years of stroke [5]. Studies have reported that the cross-sectional prevalence of PSD is 18% and 33% [5–8]. To date, tricyclic antidepressants (TCAs) and selective serotonin reuptake inhibitors (SSRIs) have been used to treat PSD. However, the lack of a timely diagnosis, the adverse effects of these drugs on cardiovascular function, and an increased risk of bleeding lead to unsatisfactory treatment for patients with PSD [9, 10].

Traditional Chinese medicine (TCM) for PSD is characterized by few side effects and individualized treatment [11]. Therefore, the search for an herbal medicine for the treatment of PSD has become a hot topic in contemporary pharmacological research. *Gan-Mai-Da-Zao* Decoction (GMDZD) is derived from *Jingui Yaolue* and is composed of three Chinese herbal medicines: *Glycyrrhiza uralensis* Fisch. (gancao in Chinese), *Triticum aestivum* L. (xiaomai in Chinese), and *Ziziphus jujuba* Mill. (dazao in Chinese); it is mainly used for sleep disorders and depression-related psychiatric disorders [12]. A randomized controlled study showed that GMDZD intervention for 2 and 4 weeks could significantly improve the Hamilton Depression Rating Scale (HAM-D) score in postpartum women in depressive states [13]. Modern pharmacological studies have also confirmed that GMDZD could increase central excitability, sedation and hypnotism, antidepressant activity, and other pharmacological activities [14–16]. However, the mechanism by which the active ingredients in GMDZD alleviate depressive symptoms is unclear.

**Figure 1:**
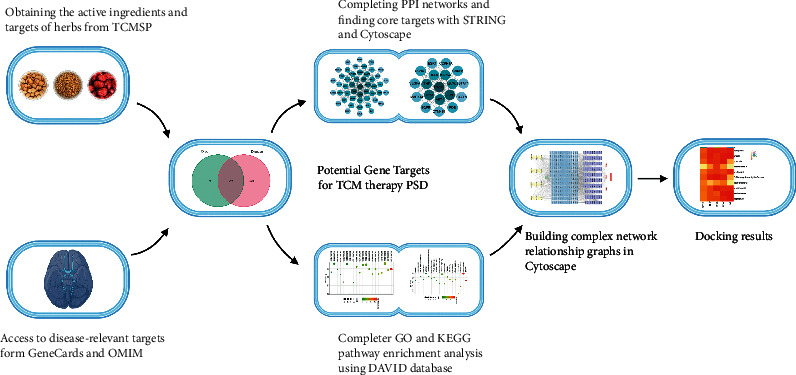
Detailed process of research design.

TCM with multiple components, multiple targets, and multiple pathways can bring very many new possibilities for clinical treatment. Network pharmacology is the result of the integration of multidisciplinary basic theories, such as those from biology, computer science, multidirectional pharmacology, molecular pharmacology, and medicine, and research tools that can systematically and comprehensively reflect the intervention mechanisms of drugs through disease networks. This has strong convergence with the principle of the overall dynamics of TCM treatment for diseases and the characteristics of multicomponent, multitarget, and multipathway interactions. Therefore, network pharmacology can provide new and powerful technical support for studying the mechanism of action of TCM compounding, which can help to reveal the scientific connotation of TCM compounding, discover drug targets, and develop TCM theories [17]. This study aimed to investigate the mechanism of action of GMDZD in the treatment of PSD through network pharmacology and molecular docking technology (Figure 1).

## 2. Materials and Methods

### 2.1. Screening Active Compounds and Predicting Putative Targets

Through the Traditional Chinese Medicine Systems Pharmacology Database and Analysis Platform (TCMSP, https://tcmspw.com/tcmsp.php), an authoritative public database that contains a large number of active ingredients, their related targets, and pharmacokinetic information [18], we searched for the active ingredients of three Chinese medicines and supplemented them with the published literature [19, 20]. Oral bioavailability (OB) is an important indicator used to evaluate the rate and extent of drug absorption into the human circulation, and drug-likeness (DL) is an indicator used to evaluate the similarity of a compound to a known drug [21, 22]. Based on these two absorption, distribution, metabolism, and excretion (ADME) mode values, we performed a preliminary screen of active ingredients to obtain the active compounds and their protein targets, where OB ≥ 30% and DL ≥ 0.18% were set as the criteria [23]. After screening, to standardize protein target information, the UniProt database (The Universal Protein Resource, https://www.uniprot.org/) was utilized to standardize the protein targets on which compounds act, resulting in more comprehensive target information such as gene IDs and gene symbols.

### 2.2. Identifying Disease-Related Targets and Filtering Intersecting Targets

Using “poststroke depression” as the keyword, we mined the GeneCards database (https://www.genecards.org/) and the OMIM database (https://omim.org/) for gene targets related to PSD [24]. The targets associated with PSD were obtained by merging the targets of the two databases and removing duplicates. The common targets were then screened by using Venny 2.1 (http://bioinformatics.psb.ugent.be/webtools/Venn/), and the common targets were defined as the potential targets of GMDZD in PSD.

### 2.3. Protein-Protein Interaction (PPI) Network Construction

A PPI network was constructed by submitting common disease-drug targets to the Search Tool for the Retrieval of Interacting Genes database (STRING, https://string-db.org/) [25]. The organism species was set as “*Homo sapiens*,” and the confidence score with a correlation degree was set as ≥0.950; meanwhile, disconnected nodes were hidden. Then, the interaction information was further visually analysed by Cytoscape 3.8.2. The CytoNCA plug-in was used to analyse the topological attributes of the data submitted to Cytoscape [26, 27]. In this plug-in, betweenness centrality (BC), closeness centrality (CC), and degree centrality (DC) are used to estimate the importance of nodes in the network. The higher the quantitative value of these three numerical values, the more important the node in the network. In the PPI network, BC, CC, and DC are used as variables to screen out the core targets and build a network relationship diagram of the core targets based on the screening results.

### 2.4. Gene Ontology (GO) and Kyoto Encyclopedia of Genes and Genomes (KEGG) Pathway Enrichment Analyses

The previously obtained GMDZD targets for the treatment of PSD were imported into the DAVID database (https://david.ncifcrf.gov/summary.jsp), a repository with comprehensive annotation capability that is updated monthly with gene annotation data to analyse the biological processes and metabolic pathways in which they are enriched. We chose “*Homo sapiens*” for the species, and terms with a *p* value <0.05 were considered significant. The results were saved and visualized using Bioinformatics (http://www.bioinformatics.com.cn/) to obtain a bubble map of the results of the GO and KEGG enrichment analyses. GO enrichment analysis covers three aspects of biology: cellular component, molecular function, and biological process. KEGG enrichment analysis can also suggest the biological mechanisms of action of drugs in the human body and the pathways involved in their regulation.

### 2.5. Network Construction

(1) Cytoscape 3.8.2 was implemented to draw the disease-herb-component-target (D-H-C-T) network to visualize the relationship between GMDZD and PSD. The different colours and shapes in the diagram represent the disease, drug, component, and target, and the “edges” represent the correlation between these parameters. (2) To more visually reflect the relationships of the target genes and the enriched pathways, a more complex but more intuitive network was constructed by combining the previous network, which includes more comprehensive information on the drugs and their active ingredients, targets, and pathways.

### 2.6. Molecular Docking Prediction

#### 2.6.1. Preparation of Small-Molecule Ligands

To better evaluate the reliability of the network predictions, the core active ingredients were then molecularly docked to the core gene targets. First, small-molecule ligands were prepared by obtaining the 2D structures of their active ingredients in the *sdf* format through the PubChem database (https://pubchem.ncbi.nlm.nih.gov/) and then converting the 2D structures into 3D structures in the *mol2* format through ChemOffice software. Finally, the results were imported into AutoDockTools to convert to files into the *pdbqt* format [28].

#### 2.6.2. Preparation of Protein Receptors

First, the core gene targets were entered into the UniProt database to obtain their UniProt IDs, and the 3D structures of the core gene targets were retrieved and downloaded from the Protein Data Bank database (PDB,http://www.rcsb.org/) in the *pdb* format. PyMOL 2.3.4 software was used to dewater and deligand the proteins, the core gene targets were hydrogenated and charge calculated using AutoDockTools [29], and the results were saved in the *pdbqt* format. Upon completion, ligand-receptor molecular docking was performed using AutoDockVina. Among the existing drugs that have been used to treat PSD, SSRIs have shown high efficacy and few side effects, with sertraline and escitalopram being the representative drugs [9, 10]. Notably, to make the conclusions more objective, we molecularly docked existing drugs already used for the treatment of PSD with the potential core gene targets we screened.

## 3. Results

### 3.1. Active Compounds and Targets of Gancao, Xiaomai, and Dazao

We found a total of 442 drug-related components from the TCMSP database as well as the available literature. Then, screening was performed according to the criteria DL ≥ 0.18 and OB ≥ 30%, and the gene targets associated with each active ingredient were obtained. Finally, duplicate targets were removed after comparison and correction by the UniProt database. A total of 109 active ingredients and 239 gene targets were obtained, among which some were derived from multiple drugs, reflecting the multicomponent and multitarget characteristics of TCM. Details on the active ingredients are shown in Supplementary Materials Table S1.

### 3.2. Gene Targets in PSD

**Figure 2:**
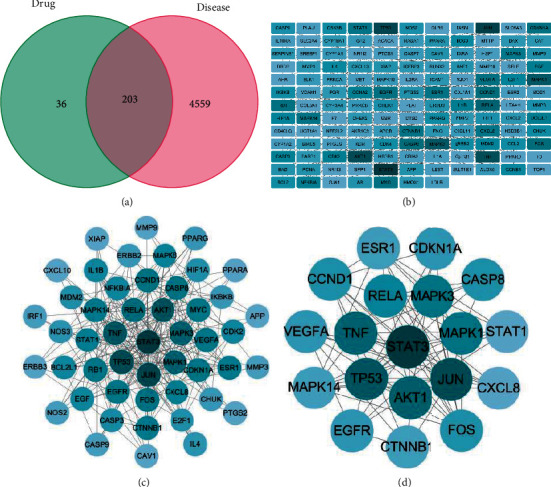
Drug-disease targets intersection Venn diagram and protein-protein interaction network. The darker the color means the larger the degree value, the more important it is in this network. (a) 203 intersection gene targets; (b) a protein-protein network from STRING; (c) one protein-protein cluster with 49 nodes and 230 edges; and (d) one protein-protein cluster with 19 nodes and 76 edges.

In this study, 4733 PSD-related gene targets were obtained from the GeneCards database, and another 37 gene targets were obtained from the OMIM database, in which there were 8 identical targets. After removing duplicates, the total number of PSD-related gene targets was 4762. Next, 203 interacting gene targets were obtained by taking the intersection of the disease-related gene targets and the drug-related gene targets (Figure 2(a)). These targets were identified as the potential targets of GMDZD against PSD (Table 1).

### 3.3. PPI Network Analysis

The 203 intersecting gene targets were submitted to the STRING database, and then, the data obtained from this platform were imported into Cytoscape 3.8.2 for visualization and topological analysis (Figure 2(b)). The PPI network consisted of 139 nodes and 426 edges. The topological properties of the intersecting gene targets were analysed using the CytoNCA plug-in, whose median BC, CC, and DC values were 22, 0.046184739, and 4, respectively. The values of 49 gene targets were above the median, and these genes were considered important for the treatment of PSD with GMDZD (Figure 2(c)). Among these genes, the values of AKT1, STAT3, TP53, CTNNB1, CDKN1A, ESR1, VEGFA, MAPK1, MAPK3, CASP8, CCND1, MAPK14, RELA, TNF, EGFR, FOS, JUN, CXCL8, and STAT1 were greater than the twofold median values of BC, CC, and DC (286.3560165, 0.046954747, and 11), indicating that these 19 genes are the core targets in the PPI network (Figure 2(d)), where STAT3 has the highest degree value, suggesting that it may be most relevant to this research. Details from the STRING analysis are shown in Supplementary Materials Table S2.

### 3.4. GO and KEGG Pathway Enrichment Analyses

The top 20 terms and pathways retrieved from the DAVID database were ranked according to their *p* value, and the data were then transformed into a bubble chart for display. The more the genes that are enriched, the larger the bubble, the deeper the colour, and the smaller the *p* value.

**Figure 3:**
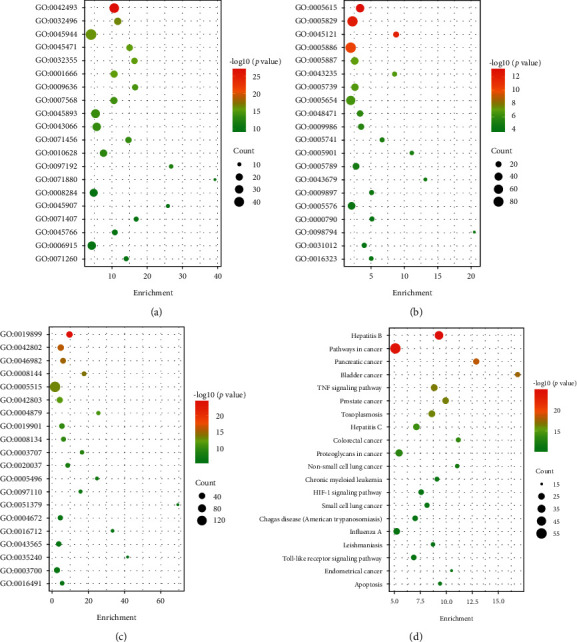
Bubble maps of GO and KEGG pathway enrichment analysis of *Gan-Mai-Da-Zao* decoction for PSD. (a) GO: biological process; (b) GO: cellular components; (c) GO: molecular functions; and (d) KEGG pathway enrichment analysis.

The main biological process terms were as follows: response to drug (GO: 0042493), response to lipopolysaccharide (GO: 0032496), positive regulation of transcription from RNA polymerase II promoter (GO: 0045944), response to ethanol (GO: 0045471), and response to estradiol (GO: 0032355) (Figure 3(a)). The main cellular component terms were as follows: extracellular space (GO: 0005615), cytosol (GO: 0005829), membrane raft (GO: 0045121), plasma membrane (GO: 0005886), and integral component of plasma membrane (GO: 0005887) (Figure 3(b)). The main molecular function terms were as follows: enzyme binding (GO: 0019899), identical protein binding (GO: 0042802), protein heterodimerization activity (GO: 0046982), drug binding (GO: 0008144), and protein binding (GO: 0005515) (Figure 3(c)). All of the abovementioned data suggest that GMDZD may treat PSD by modulating multiple GO functions.

KEGG pathway enrichment analysis yielded a total of 122 pathways, and the same criteria used for GO enrichment analysis were used to obtain the top 20 pathways for graphic visualization. The main pathways of the relevant targets in PSD were as follows: hepatitis B (hsa05161), pathways in cancer (hsa05200), pancreatic cancer (hsa05212), bladder cancer (hsa05219), and the TNF signaling pathway (hsa04668). Among these pathways, “pathways in cancer” had the highest degree of target enrichment and were, thus, identified as an important critical pathway; similarly, “hepatitis B” had the lowest *p* value and was also identified as an important pathway (Figure 3(d)). Details on the GO and KEGG enrichment analyses from the DAVID database are shown in Supplementary Materials Table S3. Supplementary Materials Table S4 shows the specific information on the targets in the top 20 pathways.

### 3.5. Network Construction

**Figure 4:**
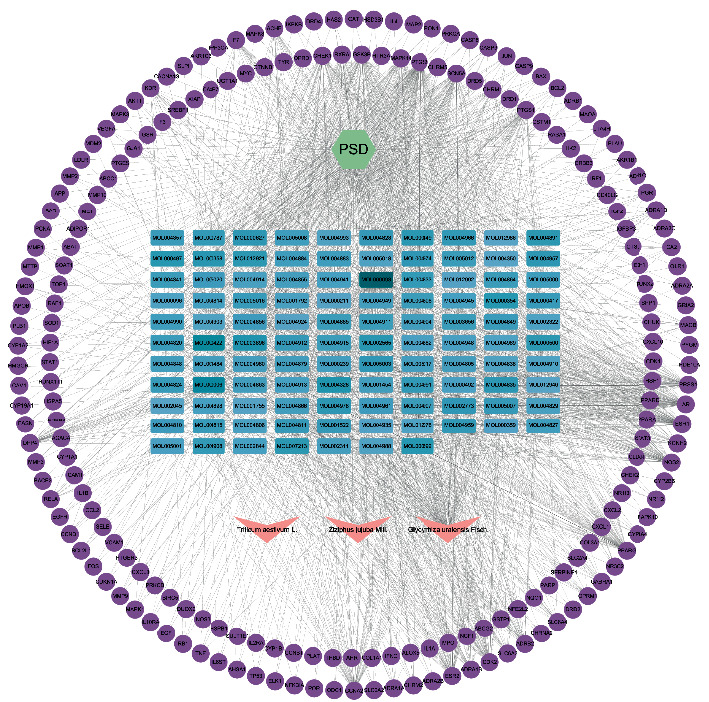
Disease-herb-compound-target (D-H-C-T) network of *Gan-Mai-Da-Zao* decoction against PSD. The purple circles represent genes, the green hexagons represent diseases, the pink V-shapes represent drugs, and finally, shades of blue rectangles represent active ingredients, and the darker the color, the greater the degree value in the network, indicating greater importance.

**Figure 5:**
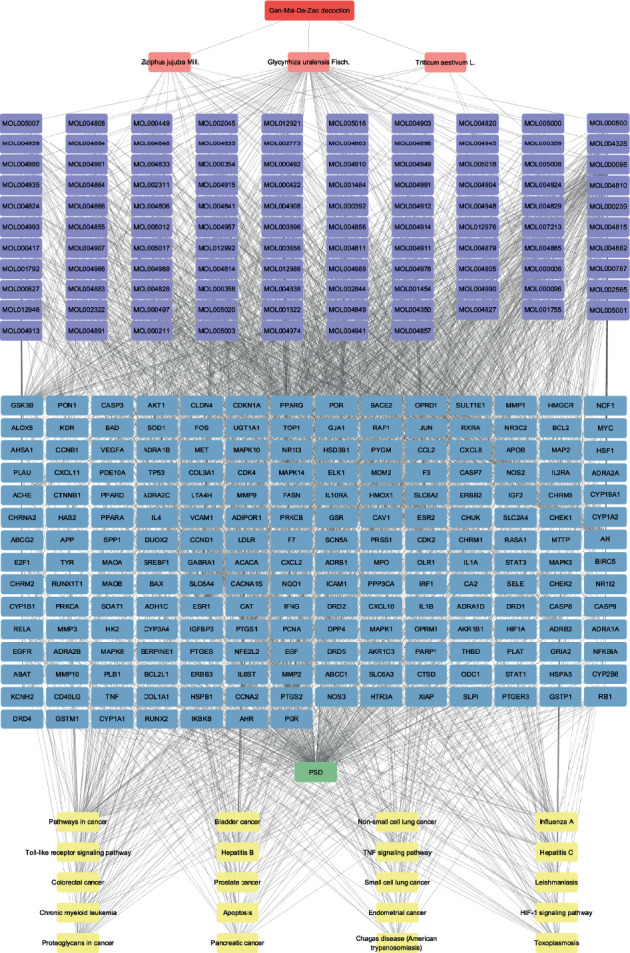
Network diagram containing pathway enrichment analysis. The different colored rectangles from top to bottom represent herbal medicine, herbs, active ingredients, targets, disease, and pathways.

There were 314 nodes and 1825 edges in the D-H-C-T network, among which the potential active ingredients icos-5-enoic acid (MOL004985) and gadelaidic acid (MOL004996) were hidden because the corresponding gene targets did not overlap with the disease targets (Figure 4). Details are shown in Supplementary Materials Table S5. The pathway information was imported into Cytoscape 3.8.2 and combined with the previous graph to obtain a new network with 335 nodes and 2300 edges (Figure 5).

### 3.6. Docking Results

**Figure 6:**
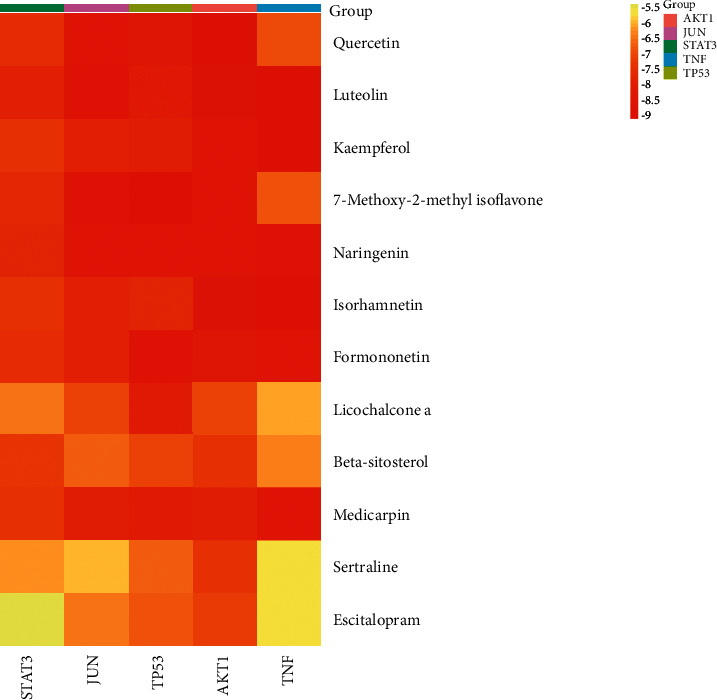
The heat map of the docking score.

**Figure 7:**
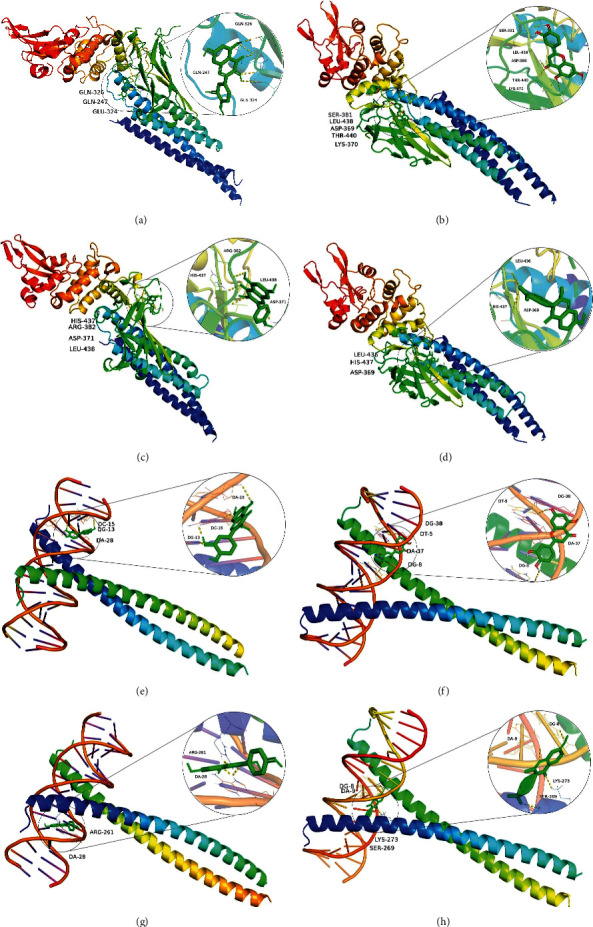
The docking complex consisting of two targets and four components. The colored irregular lines represent proteins, the green rod-like structures represent compounds, and each image shows the details of the docked parts. (a) STAT3-quercetin, (b) STAT3-luteolin, (c) STAT3-kaempferol, (d) STAT3-7-methoxy-2-methyl isoflavone, (e) JUN-quercetin, (f) JUN-luteolin, (g) JUN-kaempferol, and (h) JUN-7-methoxy-2-methyl isoflavone.

The nodes with high degree values in the PPI network were considered core gene targets, and the five core gene targets with the highest degree values in the PPI network, namely, STAT3 (PDB ID: 6NJS), JUN (PDB ID: 5T01), TP53 (PDB ID: 6WQX), AKT1 (PDB ID: 5WBL), and TNF (PDB ID: 2E7A), were correlated with the 10 active ingredients with the highest degree in the “D-H-C-T” network (quercetin (MOL000098), luteolin (MOL000006), kaempferol (MOL000422), 7-methoxy-2-methyl isoflavone (MOL003896), naringenin (MOL004328), isorhamnetin (MOL000354), formononetin (MOL000392), licochalcone a (MOL000497), beta-sitosterol (MOL000358), and medicarpin (MOL002565)). These compounds were molecularly docked, and their binding energies were calculated. On this basis, we molecularly docked sertraline and escitalopram to the core genes to refine the comparative analysis (Table 2, Figure 6). Moreover, we graphically examined the specific docking details of the top core genes (STAT3 and JUN) with the top core components (quercetin, luteolin, kaempferol, and 7-methoxy-2-methyl isoflavone) (Figure 7). The lower the binding energy of both the ligand and receptor, the more stable the binding [30]. In general, a docking fraction value of less than −4.25 kcal·mol^−1^ indicates some binding activity, a value less than −5.0 kcal·mol^−1^ indicates good binding activity, and a value less than −7.0 kcal·mol^−1^ indicates strong binding activity [31]. A total of 60 groups of core components were determined to have good binding activity with the target proteins. The comparative analysis clearly showed that sertraline and escitalopram also showed good binding activity, but some of the active ingredients screened in this study showed higher binding activity with the core gene target to some extent, which also provides direction for the development of new drugs and subsequent research.

## 4. Discussion

Network pharmacology is a method used to predict the ability of a drug to treat a certain disease by searching for shared genes and identifying enriched pathways, which are then confirmed through available experimental evidence. Molecular docking can be used to predict the binding activity of active ingredients to their target proteins, which further confirms the therapeutic effect of the drugs. The use of these techniques has largely solved the great challenges posed to research due to the multicomponent and multitarget nature of TCM. Therefore, we used network pharmacology and molecular docking to reveal the possible mechanism of action of GMDZD in PSD.

In our study, the core active ingredients in the “D-H-C-T” network were identified as quercetin, luteolin, kaempferol, naringenin, and isorhamnetin. In recent years, flavonoids have been found to have significant effects on the central nervous system, with neuroprotective, antidepressant, and anxiolytic effects [32]. In our study, quercetin was derived from *Glycyrrhiza uralensis* Fisch. and *Ziziphus jujuba* Mill. A relevant animal study confirmed that quercetin could reverse stress-induced depression and anxiety in mice [33]. In addition, quercetin exerts antidepressant effects by exerting antioxidant and anti-inflammatory activities, decreasing cytotoxicity, and increasing 5-hydroxytryptamine levels [34]. Luteolin is derived from *Triticum aestivum* L. It has been reported that luteolin exerts an antidepressant effect by suppressing the endoplasmic reticulum [35] and inhibiting and downregulating plasma membrane monoamine transporters (PMAT, Slc29a4) [36]. It has also been found that luteolin may improve cognitive performance by inhibiting microglial activation and neuroinflammation in older mice [37]. Kaempferol, naringenin, and isorhamnetin are all derived from *Glycyrrhiza uralensis* Fisch. Kaempferol promotes the protein expression of brain-derived neurotrophic factor (BDNF) and nerve growth factor (NGF) in hippocampal tissue from aged rats with chronic stress/depression, and these changes resulted in neuroprotection and improved depression-like behaviour [38]. Naringenin is a flavonoid compound with strong antioxidant and anti-inflammatory effects. The literature has shown that naringin may produce functional behavioural effects by enhancing cholinergic transmission and antioxidant defence systems and inhibiting lipid peroxidation and nitrosative processes [39]. The application of isorhamnetin potentiates nerve growth factor- (NGF-) induced neurite outgrowth. In parallel, the expression of neurofilaments is markedly increased in the cultures cotreated with NGF and isorhamnetin. This suggests that isorhamnetin might be used to some extent to treat neurodegenerative diseases, including Alzheimer's disease and depression [40]. These important active ingredients are all sourced from GMDZD, and multiple active ingredients work together to exert their effects in the treatment of PSD.

In addition, a total of 19 core targets of GMDZD in PSD were screened in the PPI network: AKT1, STAT3, TP53, CTNNB1, CDKN1A, ESR1, VEGFA, MAPK1, MAPK3, CASP8, CCND1, MAPK14, RELA, TNF, EGFR, FOS, JUN, CXCL8, and STAT1. Among them, EGFR is highly expressed in a variety of malignancies, and depression is common in oncology patients (four times more prevalent than in the general population) [41, 42]. Moreover, EGFR-mutant non-small-cell lung cancer can lead to depression by mediating inflammatory factors [43]. Depressive-like behaviour can be induced by forced swimming, and MAPK1 overexpression in the hippocampus can exert antidepressant effects [44]. It has been reported that estrogen regulates neurotransmitter conversion and thus produces antidepressant effects. It is thought that the biological function of estrogen is largely mediated by the intracellular activation of its primary receptors, estrogen receptor alpha (ESR1), and estrogen receptor beta (ESR2). Thus, genetic variation in ESR plays an important role in the susceptibility of women to depression [45, 46]. STAT3 is expressed in both hippocampal neurons and glial cells and is closely related to neurodegenerative diseases. It has been demonstrated that the pharmacological treatment of PSD and an improvement in the depressive state may be related to the inhibition of JAK2/STAT3 signaling pathway-related gene and protein expression, which promotes neural remodelling in the hippocampus [47].

Among the 20 pathways screened by KEGG enrichment analysis, some are closely related to PSD, including the TNF and toll-like receptor signaling pathways. The immune-inflammatory response is an important pathogenic mechanism of PSD. Elevated levels of various inflammatory biomarkers, such as IL-6 and TNF-*α*, and increased high-sensitivity C-reactive protein (CRP) concentrations were found to be present in patients with mild to moderate depression six months following stroke [48, 49]. It has also been shown that an improvement in depression in rats under acupuncture intervention may be closely related to the significant downregulation of differentially expressed genes involved in the toll-like receptor pathway and TNF signaling pathway in the hippocampus, frontal lobes, and pituitary gland [50]. Additionally, pathological mechanisms, such as neuronal apoptosis and nerve growth disorders, are involved in some pathways that also play an important role in the development of PSD. The efficacy of current antidepressants has been linked to the Ras signaling pathway, which may be involved in the onset and development of depression-related disorders by indirectly affecting neurotrophic factors or directly affecting neuroplasticity [51]. Furthermore, antidepressants not only upregulate cAMP levels in receptor cells but also activate protein kinase A (PKA) to phosphorylate PKA, which then activates the cAMP-response element-binding protein (CREB) signaling pathway, altering functional protein activity and gene expression patterns to form new synapses, thus exerting antidepressant effects [52].

## 5. Conclusions

In summary, network pharmacological analysis showed that there are as many as 203 possible targets of GMDZD in the treatment of PSD. Several pathways may be very closely related to the treatment of PSD, including the TNF and toll-like receptor signaling pathways, and the 19 core gene targets screened from the PPI network are also enriched in these important pathways. Therefore, the results of this study provide evidence for follow-up research and a basis for the clinical application of GMDZD in the treatment of PSD [53].

## Figures and Tables

**Table 1:** Potential targets of *Gan-Mai-Da-Zao* decoction against poststroke depression (PSD).

No.	Gene	No.	Gene	No.	Gene	No.	Gene	No.	Gene
1	PTGS1	42	PLAU	83	CDKN1A	124	THBD	165	CCNA2
2	DRD1	43	LTA4H	84	MMP9	125	SERPINE1	166	ESR2
3	CHRM1	44	MAOA	85	MAPK1	126	COL1A1	167	CDK2
4	DRD5	45	ADRB1	86	IL10RA	127	IFNG	168	MAPK10
5	SCN5A	46	BCL2	87	EGF	128	ALOX5	169	PYGM
6	CHRM5	47	BAX	88	RB1	129	IL1A	170	GRIA2
7	PTGS2	48	CASP9	89	TNF	130	MPO	171	OLR1
8	HTR3A	49	JUN	90	IL6ST	131	NCF1	172	IL4
9	RXRA	50	CASP3	91	AHSA1	132	ABCG2	173	HSD3B1
10	OPRD1	51	CASP8	92	TP53	133	GSTP1	174	IKBKB
11	SLC6A2	52	PRKCA	93	ELK1	134	NFE2L2	175	MAPK8
12	ADRA1A	53	PON1	94	NFKBIA	135	NQO1	176	PPP3CA
13	CHRM2	54	MAP2	95	POR	136	PARP1	177	AKR1C3
14	ADRA2B	55	CAT	96	ODC1	137	AHR	178	SLPI
15	ADRA1B	56	HAS2	97	TOP1	138	SLC2A4	179	MAPK3
16	SLC6A3	57	DRD4	98	RAF1	139	COL3A1	180	LDLR
17	ADRB2	58	ACHE	99	SOD1	140	CXCL11	181	BAD
18	CHRNA2	59	F7	100	HIF1A	141	CXCL2	182	MTTP
19	SLC6A4	60	CACNA1S	101	STAT1	142	NR1I3	183	APOB
20	DRD2	61	KDR	102	RUNX1T1	143	CHEK2	184	PLB1
21	OPRM1	62	AKT1	103	HSPA5	144	CLDN4	185	HMGCR
22	GABRA1	63	VEGFA	104	ERBB2	145	PPARA	186	CYP19A1
23	NR3C2	64	MMP2	105	ACACA	146	PPARD	187	UGT1A1
24	PPARG	65	MMP1	106	CYP1A1	147	HSF1	188	SREBF1
25	CYP3A4	66	HMOX1	107	ICAM1	148	CXCL10	189	GSR
26	NR1I2	67	CYP1A2	108	IL1B	149	CHUK	190	ABCC1
27	CYP2B6	68	CAV1	109	CCL2	150	SPP1	191	ADIPOR1
28	NOS2	69	CTNNB1	110	SELE	151	RUNX2	192	ABAT
29	KCNH2	70	MYC	111	VCAM1	152	E2F1	193	SOAT1
30	ESR1	71	CASP7	112	PTGER3	153	CTSD	194	BACE2
31	AR	72	F3	113	CXCL8	154	IGFBP3	195	STAT3
32	PRSS1	73	GJA1	114	PRKCB	155	IGF2	196	CDK4
33	PDE10A	74	MMP10	115	BIRC5	156	CD40LG	197	MDM2
34	MAOB	75	FASN	116	DUOX2	157	IRF1	198	APP
35	ADRA2A	76	DPP4	117	NOS3	158	ERBB3	199	PCNA
36	CA2	77	MMP3	118	HSPB1	159	HK2	200	TYR
37	ADRA2C	78	RELA	119	SULT1E1	160	RASA1	201	XIAP
38	ADRA1D	79	EGFR	120	IL2RA	161	GSTM1	202	PTGES
39	PGR	80	CCND1	121	CYP1B1	162	MAPK14	203	MET
40	ADH1C	81	BCL2L1	122	CCNB1	163	GSK3B		
41	AKR1B1	82	FOS	123	PLAT	164	CHEK1		

**Table 2:** Binding energies of 10 main compounds and 2 drugs that have been used to treat PSD to 5 potential targets.

No.	Compound	Binding energy/kcal·mol^−1^				
		STAT3	JUN	TP53	AKT1	TNF
1	Quercetin	−7.5	−8.4	−8.3	−8.9	−6.8
2	Luteolin	−7.8	−8.6	−8.2	−8.7	−8.9
3	Kaempferol	−7.3	−7.8	−7.9	−8.4	−8.9
4	7-Methoxy-2-methyl isoflavone	−7.6	−8.6	−9.0	−8.4	−6.7
5	Naringenin	−7.7	−8.5	−8.5	−8.4	−8.6
6	Isorhamnetin	−7.3	−7.8	−7.7	−8.7	−9.0
7	Formononetin	−7.4	−7.8	−8.6	−8.3	−8.4
8	Licochalcone a	−6.3	−7.0	−8.1	−7.0	−6.0
9	beta-Sitosterol	−7.2	−6.6	−7.0	−7.3	−6.2
10	Medicarpin	−7.3	−8.0	−8.1	−7.9	−8.4
11	Sertraline	−6.1	−5.9	−6.6	−7.3	−5.6
12	Escitalopram	−5.3	−6.4	−6.7	−7.1	−5.6

## Data Availability

All data are available in the manuscript, and they are exhibited in figures and tables.

## Abbreviations

PSD: Poststroke depression
GMDZD: *Gan-Mai-Da-Zao* decoction
TCAs: Tricyclic antidepressants
SSRIs: Selective serotonin reuptake inhibitors
TCM: Traditional Chinese medicine
HAM-D: Hamilton Depression Rating Scale
TCMSP: Traditional Chinese Medicine Systems Pharmacology Database and Analysis Platform
OB: Oral bioavailability
DL: Drug-likeness
ADME: Absorption, distribution, metabolism, and excretion
UniPort database: The Universal Protein Resource
PPI: Protein-protein interaction
STRING: Search Tool for the Retrieval of Interacting Genes
BC: Betweenness centrality
CC: Closeness centrality
DC: Degree centrality
GO: Gene Ontology
KEGG: Kyoto Encyclopedia of Genes and Genomes
D-H-C-T: Disease-herb-component-target
PDB: Protein Data Bank
CRP: C-reactive protein.

## Supplementary Materials

Supplementary Materials Supplementary Table S1: active compounds of iujube-licorice-wheat. Table S2: network topology parameters of gene targets in PPI. Table S3: details in GO and KEGG pathway enrichment analysis. Table S4: the specific targets information in the top 20 pathways. Table S5: the value of the network topology parameters of the active ingredient.

## References

[1] Shi Y., Yang D., Zeng Y., Wu W. (2017). Risk factors for post-stroke depression: a meta-analysis. *Frontiers in Aging Neuroscience*.

[2] Martin T. R., Oyiza M., Luis A., Evans J. J., Stott D. J., Quinn T. J. (2019). Prevalence of pre-stroke depression and its association with post-stroke depression: a systematic review and meta-analysis. *Psychological Medicine*.

[3] Zhang T. (2012). Stroke rehabilitation treatment guidelines in China. *Chinese Journal of the Frontiers of Medical Science (Electronic Version)*.

[4] Benjamin E. J., Muntner P., Alonso A. (2019). Heart disease and stroke statistics-2019 update: a report from the American heart association. *Circulation*.

[5] Ayerbe L., Ayis S., Wolfe C. D. A., Rudd A. G. (2013). Natural history, predictors and outcomes of depression after stroke: systematic review and meta-analysis. *British Journal of Psychiatry*.

[6] Hackett M. L., Pickles K. (2014). Part I: frequency of depression after stroke: an updated systematic review and meta-analysis of observational studies. *International Journal of Stroke*.

[7] Jørgensen T. S., Wium-Andersen I. K., Wium-Andersen M. K. (2016). Incidence of depression after stroke, and associated risk factors and mortality outcomes, in a large cohort of danish patients. *JAMA Psychiatry*.

[8] Mitchell A. J., Sheth B., Gill J. (2017). Prevalence and predictors of post-stroke mood disorders: a meta-analysis and meta-regression of depression, anxiety and adjustment disorder. *General Hospital Psychiatry*.

[9] Hackett M. L., Anderson C. S., House A., Xia J (2008). Interventions for treating depression after stroke. *Cochrane Database of Systematic Reviews*.

[10] Coupland C., Dhiman P., Morriss R., Arthur A., Barton G., Hippisley-Cox J. (2011). Antidepressant use and risk of adverse outcomes in older people: population based cohort study. *BMJ*.

[11] Liu S., Yang Z. H., Zhu X. N. (2021). Research progress of TCM therapies in treating post-stroke depression. *Acta Chinese Medicine and Pharmacology*.

[12] Jiang D. Y., Ren P. P., Li W. H. (2020). Study and analysis of hysteria. *Jilin Journal of Chinese Medicine*.

[13] Shao Y. (2016). The effect of adding and subtracting Gan-Mai-Da-Zao decoction on postpartum women’s sleep disorder and depressive state. *Chinese Journal of Clinical Rational Drug Use*.

[14] Xia B. M., Zhang H. L., Xue W. D. (2015). Postpartum depression animal model in mice and effect of Yuejuganmaidazaotang on PPD model. *Chinese Pharmacological Bulletin*.

[15] Xiang Y., Meng P., Zhang X. L. (2016). Research on central excitatory pharmacological effects of Ganmaidazao decoction. *Asia-Pacific Traditional Medicine*.

[16] Feng D. D., Tang T., Lin X. P. (2016). Nine traditional Chinese herbal formulas for the treatment of depression: an ethnopharmacology, phytochemistry, and pharmacology review. *Neuropsychiatric Disease and Treatment*.

[17] Chen H. B., Zhou H. G., Li W. T. (2019). Network pharmacology: a new perspective of mechanism research of traditional Chinese medicine formula. *China Journal of Traditional Chinese Medicine and Pharmacy*.

[18] Ru J., Li P., Wang J. (2014). TCMSP: a database of systems pharmacology for drug discovery from herbal medicines. *Journal of Cheminformatics*.

[19] Shan Y., Feng X., Dong Y. F. The research of chemical constituents and pharmacological activities of *Triticum* L..

[20] Zhang Y., Li Y., Wang J., Yang Y.-L., Yuan L.-J. (2014). Advance in studies on effective components in wheat bran and their pharmacological activities. *China Journal of Chinese Materia Medica*.

[21] Alam M. A., Al-Jenoobi F. I., Al-Mohizea A. M., Ali R. (2015). Understanding and managing oral bioavailability: physiological concepts and patents. *Recent Patents on Anti-Cancer Drug Discovery*.

[22] Tian S., Wang J., Li Y., Li D., Xu L., Hou T. (2015). The application of in silico drug-likeness predictions in pharmaceutical research. *Advanced Drug Delivery Reviews*.

[23] Cui Q., Zhang Y.-l., Ma Y.-h. (2020). A network pharmacology approach to investigate the mechanism of Shuxuening injection in the treatment of ischemic stroke. *Journal of Ethnopharmacology*.

[24] Fishilevich S., Zimmerman S., Kohn A. (2016). Genic insights from integrated human proteomics in GeneCards. *Database*.

[25] Szklarczyk D., Gable A. L., Lyon D. (2019). STRING v11: protein-protein association networks with increased coverage, supporting functional discovery in genome-wide experimental datasets. *Nucleic Acids Research*.

[26] Tang Y., Li M., Wang J., Pan Y., Wu F.-X. (2015). CytoNCA: a cytoscape plugin for centrality analysis and evaluation of protein interaction networks. *Biosystems*.

[27] Kohl M., Wiese S., Warscheid B. (2011). Cytoscape: software for visualization and analysis of biological networks. *Methods in Molecular Biology*.

[28] Hsin K.-Y., Matsuoka Y., Asai Y. (2016). systemsDock: a web server for network pharmacology-based prediction and analysis. *Nucleic Acids Research*.

[29] Seeliger D., de Groot B. L. (2010). Ligand docking and binding site analysis with PyMOL and autodock/vina. *Journal of Computer-Aided Molecular Design*.

[30] Trott O., Olson A. J., Vina A.D. (2010). Improving the speed and accuracy of docking with a new scoring function, efficient optimization, and multithreading. *Journal of Computational Chemistry*.

[31] Stevens W., Peneva D., Li J. Z. (2016). Estimating the future burden of cardiovascular disease and the value of lipid and blood pressure control therapies in China. *BMC Health Services Research*.

[32] Gong J. Y., Wu X. Q., Mao J. W. (2011). Advanced in studies on antidepressant effect of flavonoids. *Chinese Traditional and Herbal Drugs*.

[33] Samad N., Saleem A., Yasmin F., Shehzad M. A. (2018). Quercetin protects against stress-induced anxiety-and depression-like behavior and improves memory in male mice. *Physiological Research*.

[34] Ma N., Li Y. J., Fan J. P. (2018). Research progress on pharmacological action of quercetin. *Journal of Liaoning University of Traditional Chinese Medicine*.

[35] Ishisaka M., Kakefuda K., Yamauchi M. (2011). Luteolin shows an antidepressant-like effect via suppressing endoplasmic reticulum stress. *Biological and Pharmaceutical Bulletin*.

[36] Zhu S., Lei S., Zhou S. (2019). Luteolin shows antidepressant-like effect by inhibiting and downregulating plasma membrane monoamine transporter (PMAT, Slc29a4). *Journal of Functional Foods*.

[37] Burton M. D., Rytych J. L., Amin R., Johnson R. W. (2016). Dietary luteolin reduces proinflammatory microglia in the brain of senescent mice. *Rejuvenation Research*.

[38] Liang Y. D., Tan Y. G., Zhang S. (2020). Effect and mechanism of kaempferol on depression-like behavior in elderly rats with chronic stress depression. *The Chinese Journal of Clinical Pharmacology*.

[39] Ben-Azu B., Nwoke E. E., Aderibigbe A. O. (2019). Possible neuroprotective mechanisms of action involved in the neurobehavioral property of naringin in mice. *Biomedicine & Pharmacotherapy*.

[40] Xu S. L., Choi R. C., Zhu K. Y. (2012). Isorhamnetin, A flavonol aglycone from *Ginkgo biloba* L., induces neuronal differentiation of cultured PC12 cells: potentiating the effect of nerve growth factor. *Evidence-based Complementary and Alternative Medicine: ECAM*.

[41] Bortolato B., Hyphantis T. N., Valpione S. (2017). Depression in cancer: the many biobehavioral pathways driving tumor progression. *Cancer Treatment Reviews*.

[42] Szelei A., Döme P. (2020). Daganatos megbetegedések és a depresszió: rövid irodalmi áttekintés. *Orvosi Hetilap*.

[43] McFarland D. C., Jutagir D. R., Rosenfeld B. (2019). Depression and inflammation among epidermal growth factor receptor (EGFR) mutant nonsmall cell lung cancer patients. *Psycho-Oncology*.

[44] Iñiguez S. D., Parise L. F., Lobo M. K. (2019). Upregulation of hippocampal extracellular signal-regulated kinase (ERK)-2 induces antidepressant-like behavior in the rat forced swim test. *Behavioral Neuroscience*.

[45] Różycka A., Słopień R., Słopień A. (2016). The MAOA, COMT, MTHFR and ESR1 gene polymorphisms are associated with the risk of depression in menopausal women. *Maturitas*.

[46] Tan E.-C., Lim H.-W., Chua T.-E., Tan H.-S., Lee T., Chen H. (2018). Investigation of variants in estrogen receptor genes and perinatal depression. *Neuropsychiatric Disease and Treatment*.

[47] Liu H. P. (2020). The effect of YinaoJieyu prescription on the intervention of JAK2 and STAT3 proteins in the hippocampus of post-stroke depressed rats.

[48] Shinar D., Gross C. R., Price T. R., Banko M., Bolduc P. L., Robinson R. G. (1986). Screening for depression in stroke patients: the reliability and validity of the center for epidemiologic studies depression scale. *Stroke*.

[49] Zhang W., Zhang X.-A. (2015). A novel urinary metabolite signature for non-invasive post-stroke depression diagnosis. *Cell Biochemistry and Biophysics*.

[50] Wang Y. (2017). Study on the antidepressant mechanism of acupuncture based on whole-transcriptome sequencing.

[51] Wang F., Zhang C., Fang Y. R. (2015). Ras signaling pathway and neural plasticity mechanism of depression. *Journal of Shanghai Jiaotong University*.

[52] Wang J. M., Yang Y., Liu X. Y., Chen C. L. (2021). Research progress on mechanism and signaling pathway regulation of Chinese materia medica on depression. *Chinese Archives of Traditional Chinese Medicine*.

[53] Ding Z. C., Xu F. F., Sun Q. D. (2021). Exploring the mechanism of action of herbal medicine (Gan-Mai-Da-Zao decoction) for post-stroke depression based on network pharmacology and molecular docking. https://www.researchsquare.com/article/rs-508953/v1.

