# Supplementary material for: Exploring the Mechanism of Action of Herbal Medicine (Gan-Mai-Da-Zao Decoction) for Poststroke Depression Based on Network Pharmacology and Molecular Docking
Source: Evid Based Complement Alternat Med. 2021 Aug 21;2021:2126967. doi: 10.1155/2021/2126967 (PMC8405290; doi:10.1155/2021/2126967)
Supplement: Supplementary Materials — Supplementary Table S1: active compounds of iujube-licorice-wheat. Table S2: network topology parameters of gene targets in PPI. Table S3: details in GO and KEGG pathway enrichment analysis. Table S4: the specific targets information in the top 20 pathways. Table S5: the value of the network topology parameters of the active ingredient. [file 2126967.f1.docx]

**Supplementary Material**

**Table S1 Active compounds of Dazao-Gancao-Xiaomai**

| NO. | Mol ID | Molecule Name | OB (%) | DL | Drug |
| --- | --- | --- | --- | --- | --- |
| 1 | MOL000096 | (-)-catechin | 49.68 | 0.24 | Dazao |
| 2 | MOL000358 | beta-sitosterol | 36.91 | 0.75 | Dazao |
| 3 | MOL000492 | (+)-catechin | 54.83 | 0.24 | Dazao |
| 4 | MOL000627 | Stepholidine | 33.11 | 0.54 | Dazao |
| 5 | MOL000787 | Fumarine | 59.26 | 0.83 | Dazao |
| 6 | MOL001454 | berberine | 36.86 | 0.78 | Dazao |
| 7 | MOL001522 | (S)-Coclaurine | 42.35 | 0.24 | Dazao |
| 8 | MOL002773 | beta-carotene | 37.18 | 0.58 | Dazao |
| 9 | MOL004350 | Ruvoside_qt | 36.12 | 0.76 | Dazao |
| 10 | MOL007213 | Nuciferin | 34.43 | 0.40 | Dazao |
| 11 | MOL012921 | stepharine | 31.55 | 0.33 | Dazao |
| 12 | MOL012946 | zizyphus saponin I_qt | 32.69 | 0.62 | Dazao |
| 13 | MOL012976 | coumestrol | 32.49 | 0.34 | Dazao |
| 14 | MOL012986 | Jujubasaponin V_qt | 36.99 | 0.63 | Dazao |
| 15 | MOL012992 | Mauritine D | 89.13 | 0.45 | Dazao |
| 16 | MOL000239 | Jaranol | 50.83 | 0.29 | Gancao |
| 17 | MOL000354 | isorhamnetin | 49.60 | 0.31 | Gancao |
| 18 | MOL000392 | formononetin | 69.67 | 0.21 | Gancao |
| 19 | MOL000417 | Calycosin | 47.75 | 0.24 | Gancao |
| 20 | MOL000422 | kaempferol | 41.88 | 0.24 | Gancao |
| 21 | MOL000497 | licochalcone a | 40.79 | 0.29 | Gancao |
| 22 | MOL000500 | Vestitol | 74.66 | 0.21 | Gancao |
| 23 | MOL001484 | Inermine | 75.18 | 0.54 | Gancao |
| 24 | MOL001792 | DFV | 32.76 | 0.18 | Gancao |
| 25 | MOL002311 | Glycyrol | 90.78 | 0.67 | Gancao |
| 26 | MOL002565 | Medicarpin | 49.22 | 0.34 | Gancao |
| 27 | MOL002844 | Pinocembrin | 64.72 | 0.18 | Gancao |
| 28 | MOL003656 | Lupiwighteone | 51.64 | 0.37 | Gancao |
| 29 | MOL003896 | 7-Methoxy-2-methyl isoflavone | 42.56 | 0.20 | Gancao |
| 30 | MOL004328 | naringenin | 59.29 | 0.21 | Gancao |
| 31 | MOL004805 | (2S)-2-[4-hydroxy-3-(3-methylbut-2-enyl)phenyl]-8,8-dimethyl-2,3-dihydropyrano[2,3-f]chromen-4-one | 31.79 | 0.72 | Gancao |
| 32 | MOL004806 | euchrenone | 30.29 | 0.57 | Gancao |
| 33 | MOL004808 | glyasperin B | 65.22 | 0.44 | Gancao |
| 34 | MOL004810 | glyasperin F | 75.84 | 0.54 | Gancao |
| 35 | MOL004811 | Glyasperin C | 45.56 | 0.40 | Gancao |
| 36 | MOL004814 | Isotrifoliol | 31.94 | 0.42 | Gancao |
| 37 | MOL004815 | (E)-1-(2,4-dihydroxyphenyl)-3-(2,2-dimethylchromen-6-yl)prop-2-en-1-one | 39.62 | 0.35 | Gancao |
| 38 | MOL004820 | kanzonols W | 50.48 | 0.52 | Gancao |
| 39 | MOL004824 | (2S)-6-(2,4-dihydroxyphenyl)-2-(2-hydroxypropan-2-yl)-4-methoxy-2,3-dihydrofuro[3,2-g]chromen-7-one | 60.25 | 0.63 | Gancao |
| 40 | MOL004827 | Semilicoisoflavone B | 48.78 | 0.55 | Gancao |
| 41 | MOL004828 | Glepidotin A | 44.72 | 0.35 | Gancao |
| 42 | MOL004829 | Glepidotin B | 64.46 | 0.34 | Gancao |
| 43 | MOL004833 | Phaseolinisoflavan | 32.01 | 0.45 | Gancao |
| 44 | MOL004835 | Glypallichalcone | 61.60 | 0.19 | Gancao |
| 45 | MOL004838 | 8-(6-hydroxy-2-benzofuranyl)-2,2-dimethyl-5-chromenol | 58.44 | 0.38 | Gancao |
| 46 | MOL004841 | Licochalcone B | 76.76 | 0.19 | Gancao |
| 47 | MOL004848 | licochalcone G | 49.25 | 0.32 | Gancao |
| 48 | MOL004849 | 3-(2,4-dihydroxyphenyl)-8-(1,1-dimethylprop-2-enyl)-7-hydroxy-5-methoxy-coumarin | 59.62 | 0.43 | Gancao |
| 49 | MOL004855 | Licoricone | 63.58 | 0.47 | Gancao |
| 50 | MOL004856 | Gancaonin A | 51.08 | 0.40 | Gancao |
| 51 | MOL004857 | Gancaonin B | 48.79 | 0.45 | Gancao |
| 52 | MOL004863 | 3-(3,4-dihydroxyphenyl)-5,7-dihydroxy-8-(3-methylbut-2-enyl)chromone | 66.37 | 0.41 | Gancao |
| 53 | MOL004864 | 5,7-dihydroxy-3-(4-methoxyphenyl)-8-(3-methylbut-2-enyl)chromone | 30.49 | 0.41 | Gancao |
| 54 | MOL004866 | 2-(3,4-dihydroxyphenyl)-5,7-dihydroxy-6-(3-methylbut-2-enyl)chromone | 44.15 | 0.41 | Gancao |
| 55 | MOL004879 | Glycyrin | 52.61 | 0.47 | Gancao |
| 56 | MOL004882 | Licocoumarone | 33.21 | 0.36 | Gancao |
| 57 | MOL004883 | Licoisoflavone | 41.61 | 0.42 | Gancao |
| 58 | MOL004884 | Licoisoflavone B | 38.93 | 0.55 | Gancao |
| 59 | MOL004885 | licoisoflavanone | 52.47 | 0.54 | Gancao |
| 60 | MOL004891 | shinpterocarpin | 80.30 | 0.73 | Gancao |
| 61 | MOL004898 | (E)-3-[3,4-dihydroxy-5-(3-methylbut-2-enyl)phenyl]-1-(2,4-dihydroxyphenyl)prop-2-en-1-one | 46.27 | 0.31 | Gancao |
| 62 | MOL004903 | liquiritin | 65.69 | 0.74 | Gancao |
| 63 | MOL004904 | licopyranocoumarin | 80.36 | 0.65 | Gancao |
| 64 | MOL004907 | Glyzaglabrin | 61.07 | 0.35 | Gancao |
| 65 | MOL004908 | Glabridin | 53.25 | 0.47 | Gancao |
| 66 | MOL004910 | Glabranin | 52.90 | 0.31 | Gancao |
| 67 | MOL004911 | Glabrene | 46.27 | 0.44 | Gancao |
| 68 | MOL004912 | Glabrone | 52.51 | 0.50 | Gancao |
| 69 | MOL004913 | 1,3-dihydroxy-9-methoxy-6-benzofurano[3,2-c]chromenone | 48.14 | 0.43 | Gancao |
| 70 | MOL004914 | 1,3-dihydroxy-8,9-dimethoxy-6-benzofurano[3,2-c]chromenone | 62.90 | 0.53 | Gancao |
| 71 | MOL004915 | Eurycarpin A | 43.28 | 0.37 | Gancao |
| 72 | MOL004924 | (-)-Medicocarpin | 40.99 | 0.95 | Gancao |
| 73 | MOL004935 | Sigmoidin-B | 34.88 | 0.41 | Gancao |
| 74 | MOL004941 | (2R)-7-hydroxy-2-(4-hydroxyphenyl)chroman-4-one | 71.12 | 0.18 | Gancao |
| 75 | MOL004945 | (2S)-7-hydroxy-2-(4-hydroxyphenyl)-8-(3-methylbut-2-enyl)chroman-4-one | 36.57 | 0.32 | Gancao |
| 76 | MOL004948 | Isoglycyrol | 44.70 | 0.84 | Gancao |
| 77 | MOL004949 | Isolicoflavonol | 45.17 | 0.42 | Gancao |
| 78 | MOL004957 | HMO | 38.37 | 0.21 | Gancao |
| 79 | MOL004959 | 1-Methoxyphaseollidin | 69.98 | 0.64 | Gancao |
| 80 | MOL004961 | Quercetin der. | 46.45 | 0.33 | Gancao |
| 81 | MOL004966 | 3'-Hydroxy-4'-O-Methylglabridin | 43.71 | 0.57 | Gancao |
| 82 | MOL004974 | 3'-Methoxyglabridin | 46.16 | 0.57 | Gancao |
| 83 | MOL004978 | 2-[(3R)-8,8-dimethyl-3,4-dihydro-2H-pyrano[6,5-f]chromen-3-yl]-5-methoxyphenol | 36.21 | 0.52 | Gancao |
| 84 | MOL004980 | Inflacoumarin A | 39.71 | 0.33 | Gancao |
| 85 | MOL004985 | icos-5-enoic acid | 30.70 | 0.20 | Gancao |
| 86 | MOL004988 | Kanzonol F | 32.47 | 0.89 | Gancao |
| 87 | MOL004989 | 6-prenylated eriodictyol | 39.22 | 0.41 | Gancao |
| 88 | MOL004990 | 7,2',4'-trihydroxy－5-methoxy-3－arylcoumarin | 83.71 | 0.27 | Gancao |
| 89 | MOL004991 | 7-Acetoxy-2-methylisoflavone | 38.92 | 0.26 | Gancao |
| 90 | MOL004993 | 8-prenylated eriodictyol | 53.79 | 0.40 | Gancao |
| 91 | MOL004996 | gadelaidic acid | 30.70 | 0.20 | Gancao |
| 92 | MOL005000 | Gancaonin G | 60.44 | 0.39 | Gancao |
| 93 | MOL005001 | Gancaonin H | 50.10 | 0.78 | Gancao |
| 94 | MOL005003 | Licoagrocarpin | 58.81 | 0.58 | Gancao |
| 95 | MOL005007 | Glyasperins M | 72.67 | 0.59 | Gancao |
| 96 | MOL005008 | Glycyrrhiza flavonol A | 41.28 | 0.60 | Gancao |
| 97 | MOL005012 | Licoagroisoflavone | 57.28 | 0.49 | Gancao |
| 98 | MOL005016 | Odoratin | 49.95 | 0.30 | Gancao |
| 99 | MOL005017 | Phaseol | 78.77 | 0.58 | Gancao |
| 100 | MOL005018 | Xambioona | 54.85 | 0.87 | Gancao |
| 101 | MOL005020 | dehydroglyasperins C | 53.82 | 0.37 | Gancao |
| 102 | MOL000006 | luteolin | 36.16 | 0.25 | Xiaomai |
| 103 | MOL001755 | 24-Ethylcholest-4-en-3-one | 36.08 | 0.76 | Xiaomai |
| 104 | MOL002045 | Stigmasterol | 43.41 | 0.76 | Xiaomai |
| 105 | MOL002322 | isovitexin | 31.29 | 0.72 | Xiaomai |
| 106 | MOL000098 | quercetin | 46.43 | 0.28 | Dazao Gancao |
| 107 | MOL000211 | Mairin | 55.38 | 0.78 | Dazao Gancao |
| 108 | MOL000449 | Stigmasterol | 43.83 | 0.76 | Dazao Xiaomai |
| 109 | MOL000359 | sitosterol | 36.91 | 0.75 | Xiaomai Gancao |

**Table S2 Network topology parameters of gene targets in PPI**

| NO. | Target | BC | CC | DC | NO. | Target | BC | CC | DC |
| --- | --- | --- | --- | --- | --- | --- | --- | --- | --- |
| 1 | ACACA | 0 | 0.007513066 | 1 | 71 | IL4 | 96.63510367 | 0.046954747 | 8 |
| 2 | FASN | 10 | 0.007515112 | 2 | 72 | CDK4 | 12.05865984 | 0.046046046 | 7 |
| 3 | AHR | 0 | 0.007571186 | 1 | 73 | CDK2 | 58.98386707 | 0.046433378 | 11 |
| 4 | CYP1A1 | 22 | 0.007573679 | 3 | 74 | CHEK1 | 7.591753825 | 0.046046046 | 6 |
| 5 | AKR1C3 | 0 | 0.043740095 | 2 | 75 | CHEK2 | 6.24651573 | 0.045984672 | 4 |
| 6 | HSD3B1 | 0 | 0.043740095 | 2 | 76 | PCNA | 42.65909401 | 0.045938748 | 5 |
| 7 | CYP19A1 | 700 | 0.045424621 | 4 | 77 | CD40LG | 0 | 0.045634921 | 1 |
| 8 | AKT1 | 1692.255546 | 0.047684865 | 25 | 78 | NFKBIA | 213.9080856 | 0.046875 | 11 |
| 9 | CAT | 245.3783826 | 0.046107584 | 4 | 79 | PRKCB | 61.39504424 | 0.045039164 | 3 |
| 10 | HSPB1 | 2.287684538 | 0.046402152 | 3 | 80 | COL1A1 | 236 | 0.044703596 | 2 |
| 11 | RAF1 | 18.05702722 | 0.046480296 | 5 | 81 | MMP2 | 486.1374866 | 0.046449007 | 4 |
| 12 | MDM2 | 54.53001539 | 0.046543002 | 9 | 82 | COL3A1 | 0 | 0.043057722 | 1 |
| 13 | STAT3 | 3823.786593 | 0.048083624 | 34 | 83 | KDR | 2.152380952 | 0.045923461 | 3 |
| 14 | ERBB3 | 25.24981049 | 0.046433378 | 5 | 84 | ERBB2 | 52.87238265 | 0.046464646 | 7 |
| 15 | RB1 | 259.5226201 | 0.047066849 | 15 | 85 | GJA1 | 0 | 0.045335085 | 1 |
| 16 | TP53 | 1437.668758 | 0.047767394 | 27 | 86 | MET | 6.391963592 | 0.046386555 | 2 |
| 17 | SOD1 | 0 | 0.045862413 | 2 | 87 | CTSD | 0 | 0.045335085 | 1 |
| 18 | NOS3 | 147.8525272 | 0.046875 | 8 | 88 | CXCL10 | 41.38448173 | 0.046433378 | 5 |
| 19 | BCL2L1 | 197.8904313 | 0.047260274 | 11 | 89 | STAT1 | 399.1186499 | 0.047276465 | 13 |
| 20 | CASP3 | 452.7224729 | 0.046795524 | 10 | 90 | CXCL2 | 25.20033483 | 0.04629319 | 4 |
| 21 | GSK3B | 20.20877033 | 0.046843177 | 7 | 91 | CXCL11 | 0 | 0.045394737 | 3 |
| 22 | CASP9 | 74.43316402 | 0.046370968 | 6 | 92 | SULT1E1 | 0 | 0.043726236 | 1 |
| 23 | CAV1 | 40.61231545 | 0.046779661 | 5 | 93 | GSTM1 | 0 | 0.007571186 | 1 |
| 24 | CTNNB1 | 562.1697547 | 0.047163363 | 14 | 94 | CYP3A4 | 34 | 0.00757451 | 4 |
| 25 | E2F1 | 42.93663101 | 0.046339825 | 9 | 95 | CYP1A2 | 12 | 0.007572848 | 2 |
| 26 | CHUK | 156.2754034 | 0.046747967 | 7 | 96 | NR1I3 | 0 | 0.007570355 | 1 |
| 27 | BAD | 10.67406547 | 0.046231156 | 5 | 97 | NR1I2 | 0 | 0.007572016 | 1 |
| 28 | CDKN1A | 439.1354391 | 0.047373841 | 15 | 98 | UGT1A1 | 0 | 0.007572016 | 1 |
| 29 | ESR1 | 1273.741418 | 0.047163363 | 14 | 99 | DRD2 | 0 | 0.007246377 | 1 |
| 30 | IKBKB | 156.2754034 | 0.046747967 | 7 | 100 | SLC6A3 | 0 | 0.007246377 | 1 |
| 31 | HIF1A | 86.73951218 | 0.047373841 | 9 | 101 | SERPINE1 | 246.984903 | 0.045680238 | 4 |
| 32 | VEGFA | 887.9667209 | 0.047373841 | 15 | 102 | RASA1 | 6.862747254 | 0.046107584 | 3 |
| 33 | MYC | 275.9498239 | 0.047586207 | 15 | 103 | IGF2 | 11.3525234 | 0.045801527 | 3 |
| 34 | ALOX5 | 352 | 0.044732577 | 3 | 104 | PLAU | 9.572960625 | 0.045559591 | 2 |
| 35 | LTA4H | 0 | 0.043084608 | 1 | 105 | MMP3 | 358.7435537 | 0.046449007 | 6 |
| 36 | PTGS1 | 2 | 0.043111528 | 2 | 106 | PRKCA | 182.1709656 | 0.045938748 | 3 |
| 37 | PTGS2 | 922 | 0.046449007 | 5 | 107 | ELK1 | 0 | 0.046030687 | 3 |
| 38 | APOB | 18 | 0.00751634 | 3 | 108 | PGR | 23.41542805 | 0.046246649 | 4 |
| 39 | OLR1 | 0 | 0.007514293 | 1 | 109 | ESR2 | 1.289337474 | 0.045923461 | 2 |
| 40 | LDLR | 18 | 0.007516749 | 2 | 110 | F3 | 0 | 0.007246377 | 1 |
| 41 | MTTP | 0 | 0.007514293 | 1 | 111 | F7 | 0 | 0.007246377 | 1 |
| 42 | APP | 22.28137926 | 0.046262152 | 5 | 112 | SREBF1 | 16 | 0.00751634 | 2 |
| 43 | MAPK1 | 782.3403851 | 0.047651934 | 21 | 113 | PPARG | 542.4377475 | 0.046747967 | 7 |
| 44 | MAPK3 | 754.2439623 | 0.047569804 | 21 | 114 | GRIA2 | 7.607401749 | 0.044358727 | 2 |
| 45 | CASP8 | 571.1476558 | 0.047066849 | 16 | 115 | HMOX1 | 239.962822 | 0.046262152 | 4 |
| 46 | MAPK10 | 6.76270594 | 0.046231156 | 3 | 116 | NQO1 | 0 | 0.044501774 | 1 |
| 47 | MAPK8 | 212.6473005 | 0.046875 | 12 | 117 | NFE2L2 | 0 | 0.046030687 | 2 |
| 48 | AR | 15.01519836 | 0.046386555 | 5 | 118 | HSF1 | 0 | 0.04583195 | 2 |
| 49 | CCND1 | 482.1970284 | 0.04740639 | 16 | 119 | ICAM1 | 248.7869048 | 0.046558704 | 4 |
| 50 | BCL2 | 20.60343293 | 0.046464646 | 7 | 120 | IRF1 | 60.15027228 | 0.046464646 | 6 |
| 51 | BAX | 0 | 0.046061415 | 4 | 121 | SELE | 0 | 0.044776119 | 1 |
| 52 | BIRC5 | 58.36509093 | 0.046574418 | 4 | 122 | IFNG | 2.149007937 | 0.046169287 | 4 |
| 53 | CCNB1 | 15.27813853 | 0.045695364 | 8 | 123 | IGFBP3 | 73.1265915 | 0.046184739 | 4 |
| 54 | CCNA2 | 28.34681778 | 0.046184739 | 10 | 124 | MMP1 | 21.78681504 | 0.04629319 | 4 |
| 55 | MAPK14 | 512.5666069 | 0.04740639 | 14 | 125 | IL10RA | 0 | 0.04632427 | 2 |
| 56 | TOP1 | 0 | 0.044995109 | 1 | 126 | IL1A | 0 | 0.045335085 | 2 |
| 57 | PARP1 | 22.1480837 | 0.045157068 | 4 | 127 | IL2RA | 0 | 0.046184739 | 1 |
| 58 | XIAP | 52.27502924 | 0.04629319 | 5 | 128 | IL6ST | 0 | 0.04632427 | 2 |
| 59 | CASP7 | 15.38859317 | 0.045335085 | 3 | 129 | VCAM1 | 1.317460317 | 0.045665122 | 2 |
| 60 | IL1B | 324.8983306 | 0.04714725 | 11 | 130 | PPARA | 163.5509339 | 0.046339825 | 5 |
| 61 | RELA | 365.0721872 | 0.047325103 | 18 | 131 | RUNX2 | 0.983255524 | 0.046107584 | 3 |
| 62 | TNF | 1220.214678 | 0.047487956 | 24 | 132 | MMP9 | 128.9610915 | 0.046449007 | 5 |
| 63 | GSR | 0 | 0.044358727 | 1 | 133 | MMP10 | 0 | 0.044805195 | 2 |
| 64 | NOS2 | 165.3130524 | 0.046922815 | 6 | 134 | SPP1 | 0 | 0.044674652 | 1 |
| 65 | EGF | 286.3560165 | 0.046875 | 11 | 135 | PLAT | 0 | 0.043963046 | 1 |
| 66 | EGFR | 776.8561364 | 0.047341338 | 15 | 136 | RXRA | 238 | 0.045171849 | 3 |
| 67 | CCL2 | 5.268586493 | 0.046906866 | 7 | 137 | PPARD | 0 | 0.043491963 | 1 |
| 68 | FOS | 494.9482167 | 0.047553411 | 16 | 138 | SLC2A4 | 0 | 0.04495114 | 1 |
| 69 | JUN | 1631.890798 | 0.047883414 | 29 | 139 | PTGES | 116 | 0.044703596 | 2 |
| 70 | CXCL8 | 421.3057711 | 0.047179487 | 13 |  |  |  |  |  |

**Table S3 Details in GO and KEGG pathway enrichment analysis**

| NO. | Term ID | Term | Enrichment | *P*-Value | -Log10(*p*) | Count | Term Type |
| --- | --- | --- | --- | --- | --- | --- | --- |
| 1 | GO:0042493 | response to drug | 10.612 | 7.75745E-28 | 27.110 | 39 | BP |
| 2 | GO:0032496 | response to lipopolysaccharide | 11.601 | 4.20045E-17 | 16.377 | 23 | BP |
| 3 | GO:0045944 | positive regulation of transcription from RNA polymerase II promoter | 4.047 | 1.26157E-16 | 15.899 | 48 | BP |
| 4 | GO:0045471 | response to ethanol | 14.968 | 3.94052E-16 | 15.404 | 19 | BP |
| 5 | GO:0032355 | response to estradiol | 16.362 | 5.8916E-16 | 15.230 | 18 | BP |
| 6 | GO:0001666 | response to hypoxia | 10.580 | 1.67181E-15 | 14.777 | 22 | BP |
| 7 | GO:0009636 | response to toxic substance | 16.544 | 4.05312E-15 | 14.392 | 17 | BP |
| 8 | GO:0007568 | aging | 10.528 | 9.37628E-15 | 14.028 | 21 | BP |
| 9 | GO:0045893 | positive regulation of transcription, DNA-templated | 5.300 | 1.93937E-14 | 13.712 | 33 | BP |
| 10 | GO:0043066 | negative regulation of apoptotic process | 5.636 | 2.92296E-14 | 13.534 | 31 | BP |
| 11 | GO:0071456 | cellular response to hypoxia | 14.648 | 3.00624E-14 | 13.522 | 17 | BP |
| 12 | GO:0010628 | positive regulation of gene expression | 7.577 | 9.80535E-14 | 13.009 | 24 | BP |
| 13 | GO:0097192 | extrinsic apoptotic signaling pathway in absence of ligand | 26.762 | 5.1774E-12 | 11.286 | 11 | BP |
| 14 | GO:0071880 | adenylate cyclase-activating adrenergic receptor signaling pathway | 39.183 | 2.57548E-11 | 10.589 | 9 | BP |
| 15 | GO:0008284 | positive regulation of cell proliferation | 4.793 | 7.40727E-11 | 10.130 | 27 | BP |
| 16 | GO:0045907 | positive regulation of vasoconstriction | 25.850 | 9.74358E-11 | 10.011 | 10 | BP |
| 17 | GO:0071407 | cellular response to organic cyclic compound | 16.824 | 9.8228E-11 | 10.008 | 12 | BP |
| 18 | GO:0045766 | positive regulation of angiogenesis | 10.789 | 1.03703E-10 | 9.984 | 15 | BP |
| 19 | GO:0006915 | apoptotic process | 4.231 | 2.18935E-10 | 9.660 | 29 | BP |
| 20 | GO:0071260 | cellular response to mechanical stimulus | 13.981 | 7.92527E-10 | 9.101 | 12 | BP |
| 21 | GO:0005615 | extracellular space | 3.332 | 6.34882E-14 | 13.197 | 50 | CC |
| 22 | GO:0005829 | cytosol | 2.194 | 6.75684E-13 | 12.170 | 81 | CC |
| 23 | GO:0045121 | membrane raft | 8.716 | 1.58298E-12 | 11.801 | 20 | CC |
| 24 | GO:0005886 | plasma membrane | 1.917 | 6.76096E-11 | 10.170 | 88 | CC |
| 25 | GO:0005887 | integral component of plasma membrane | 2.538 | 9.45401E-08 | 7.024 | 40 | CC |
| 26 | GO:0043235 | receptor complex | 8.483 | 1.71358E-07 | 6.766 | 12 | CC |
| 27 | GO:0005739 | mitochondrion | 2.563 | 1.7388E-07 | 6.760 | 38 | CC |
| 28 | GO:0005654 | nucleoplasm | 1.935 | 3.19609E-07 | 6.495 | 60 | CC |
| 29 | GO:0048471 | perinuclear region of cytoplasm | 3.325 | 1.62951E-06 | 5.788 | 23 | CC |
| 30 | GO:0009986 | cell surface | 3.478 | 2.70529E-06 | 5.568 | 21 | CC |
| 31 | GO:0005741 | mitochondrial outer membrane | 6.628 | 6.45463E-06 | 5.190 | 11 | CC |
| 32 | GO:0005901 | caveola | 11.049 | 7.48558E-06 | 5.126 | 8 | CC |
| 33 | GO:0005789 | endoplasmic reticulum membrane | 2.708 | 1.07125E-05 | 4.970 | 26 | CC |
| 34 | GO:0043679 | axon terminus | 13.092 | 1.43382E-05 | 4.844 | 7 | CC |
| 35 | GO:0009897 | external side of plasma membrane | 5.058 | 2.65749E-05 | 4.576 | 12 | CC |
| 36 | GO:0005576 | extracellular region | 2.063 | 3.68553E-05 | 4.434 | 37 | CC |
| 37 | GO:0000790 | nuclear chromatin | 5.117 | 6.05952E-05 | 4.218 | 11 | CC |
| 38 | GO:0098794 | postsynapse | 20.403 | 9.16522E-05 | 4.038 | 5 | CC |
| 39 | GO:0031012 | extracellular matrix | 3.943 | 0.000120359 | 3.920 | 13 | CC |
| 40 | GO:0016323 | basolateral plasma membrane | 4.987 | 0.000185538 | 3.732 | 10 | CC |
| 41 | GO:0019899 | enzyme binding | 9.489 | 2.43958E-25 | 24.613 | 38 | MF |
| 42 | GO:0042802 | identical protein binding | 4.663 | 1.4127E-16 | 15.850 | 42 | MF |
| 43 | GO:0046982 | protein heterodimerization activity | 5.902 | 9.29373E-16 | 15.032 | 33 | MF |
| 44 | GO:0008144 | drug binding | 17.507 | 1.30915E-14 | 13.883 | 16 | MF |
| 45 | GO:0005515 | protein binding | 1.486 | 9.77184E-14 | 13.010 | 157 | MF |
| 46 | GO:0042803 | protein homodimerization activity | 4.101 | 1.78984E-12 | 11.747 | 36 | MF |
| 47 | GO:0004879 | RNA polymerase II transcription factor activity, ligand-activated sequence-specific DNA binding | 25.409 | 9.33603E-12 | 11.030 | 11 | MF |
| 48 | GO:0019901 | protein kinase binding | 5.308 | 1.49557E-10 | 9.825 | 24 | MF |
| 49 | GO:0008134 | transcription factor binding | 6.149 | 2.16194E-10 | 9.665 | 21 | MF |
| 50 | GO:0003707 | steroid hormone receptor activity | 16.335 | 1.06249E-09 | 8.974 | 11 | MF |
| 51 | GO:0020037 | heme binding | 8.498 | 1.02072E-08 | 7.991 | 14 | MF |
| 52 | GO:0005496 | steroid binding | 24.639 | 2.29518E-08 | 7.639 | 8 | MF |
| 53 | GO:0097110 | scaffold protein binding | 15.592 | 9.16118E-08 | 7.038 | 9 | MF |
| 54 | GO:0051379 | epinephrine binding | 69.298 | 2.93018E-07 | 6.533 | 5 | MF |
| 55 | GO:0004672 | protein kinase activity | 4.401 | 3.31464E-07 | 6.480 | 19 | MF |
| 56 | GO:0016712 | oxidoreductase activity, acting on paired donors, with incorporation or reduction of molecular oxygen, reduced flavin or flavoprotein as one donor, and incorporation of one atom of oxygen | 33.263 | 6.36074E-07 | 6.196 | 6 | MF |
| 57 | GO:0043565 | sequence-specific DNA binding | 3.532 | 1.11803E-06 | 5.952 | 22 | MF |
| 58 | GO:0035240 | dopamine binding | 41.579 | 3.95058E-06 | 5.403 | 5 | MF |
| 59 | GO:0003700 | transcription factor activity, sequence-specific DNA binding | 2.596 | 3.99109E-06 | 5.399 | 30 | MF |
| 60 | GO:0016491 | oxidoreductase activity | 5.405 | 5.31621E-06 | 5.274 | 13 | MF |
| 61 | hsa05161 | Hepatitis B | 9.287 | 2.0211E-25 | 24.694 | 37 | KEGG |
| 62 | hsa05200 | Pathways in cancer | 5.094 | 1.19109E-24 | 23.924 | 55 | KEGG |
| 63 | hsa05212 | Pancreatic cancer | 12.879 | 4.90494E-19 | 18.309 | 23 | KEGG |
| 64 | hsa05219 | Bladder cancer | 16.867 | 3.61971E-18 | 17.441 | 19 | KEGG |
| 65 | hsa04668 | TNF signaling pathway | 8.844 | 3.70001E-17 | 16.432 | 26 | KEGG |
| 66 | hsa05215 | Prostate cancer | 9.926 | 4.98647E-17 | 16.302 | 24 | KEGG |
| 67 | hsa05145 | Toxoplasmosis | 8.603 | 7.51486E-17 | 16.124 | 26 | KEGG |
| 68 | hsa05160 | Hepatitis C | 7.115 | 9.53451E-15 | 14.021 | 26 | KEGG |
| 69 | hsa05210 | Colorectal cancer | 11.154 | 2.07238E-14 | 13.684 | 19 | KEGG |
| 70 | hsa05205 | Proteoglycans in cancer | 5.460 | 7.13914E-14 | 13.146 | 30 | KEGG |
| 71 | hsa05223 | Non-small cell lung cancer | 11.049 | 8.18569E-13 | 12.087 | 17 | KEGG |
| 72 | hsa05220 | Chronic myeloid leukemia | 9.099 | 4.67723E-12 | 11.330 | 18 | KEGG |
| 73 | hsa04066 | HIF-1 signaling pathway | 7.583 | 7.13349E-12 | 11.147 | 20 | KEGG |
| 74 | hsa05222 | Small cell lung cancer | 8.136 | 7.81413E-12 | 11.107 | 19 | KEGG |
| 75 | hsa05142 | Chagas disease (American trypanosomiasis) | 6.999 | 3.15552E-11 | 10.501 | 20 | KEGG |
| 76 | hsa05164 | Influenza A | 5.229 | 3.66534E-11 | 10.436 | 25 | KEGG |
| 77 | hsa05140 | Leishmaniasis | 8.715 | 4.3951E-11 | 10.357 | 17 | KEGG |
| 78 | hsa04620 | Toll-like receptor signaling pathway | 6.867 | 4.47558E-11 | 10.349 | 20 | KEGG |
| 79 | hsa05213 | Endometrial cancer | 10.499 | 5.69657E-11 | 10.244 | 15 | KEGG |
| 80 | hsa04210 | Apoptosis | 9.393 | 6.12748E-11 | 10.213 | 16 | KEGG |

**Table S4 The specific targets information in the Top 20 pathways**

| NO. | Term ID | Term | Genes |
| --- | --- | --- | --- |
| 1 | hsa05161 | Hepatitis B | RB1, CDKN1A, PCNA, CXCL8, ELK1, TNF, RELA, CASP9, IKBKB, MAPK8, CASP8, CCND1, MYC, CASP3, E2F1, AKT1, MAPK1, MAPK3, JUN, CHUK, PRKCB, STAT1, BAD, STAT3, PRKCA, FOS, MMP9, MAPK10, NFKBIA, CCNA2, CDK4, CDK2, BCL2, BAX, BIRC5, RAF1, TP53 |
| 2 | hsa05200 | Pathways in cancer | RB1, GSK3B, CDKN1A, CXCL8, CASP9, IKBKB, CASP8, CCND1, MYC, CASP3, AKT1, CHUK, PRKCB, MMP1, MMP2, PRKCA, FOS, MMP9, AR, BIRC5, PPARG, RAF1, MET, TP53, PPARD, GSTP1, PTGER3, XIAP, PTGS2, HIF1A, EGFR, RELA, MAPK8, RXRA, ERBB2, E2F1, MAPK1, MAPK3, RUNX1T1, JUN, NOS2, EGF, BAD, STAT1, STAT3, VEGFA, NFKBIA, MAPK10, CDK4, CDK2, BCL2, MDM2, BAX, CTNNB1, BCL2L1 |
| 3 | hsa05212 | Pancreatic cancer | RB1, CHUK, STAT1, BAD, EGF, STAT3, EGFR, RELA, VEGFA, MAPK10, CASP9, IKBKB, MAPK8, CCND1, CDK4, ERBB2, E2F1, AKT1, MAPK1, RAF1, TP53, BCL2L1, MAPK3 |
| 4 | hsa05219 | Bladder cancer | RB1, CDKN1A, CXCL8, MMP1, EGF, MMP2, MMP9, EGFR, VEGFA, CCND1, CDK4, MYC, ERBB2, MDM2, E2F1, MAPK1, RAF1, TP53, MAPK3 |
| 5 | hsa04668 | TNF signaling pathway | PTGS2, TNF, CXCL2, RELA, ICAM1, IKBKB, CASP7, MAPK8, CASP8, CASP3, CCL2, AKT1, MAPK1, MAPK3, JUN, VCAM1, CHUK, MMP3, FOS, MAPK14, SELE, MMP9, MAPK10, NFKBIA, CXCL10, IL1B |
| 6 | hsa05215 | Prostate cancer | RB1, GSK3B, CDKN1A, CHUK, BAD, EGF, EGFR, RELA, CASP9, NFKBIA, IKBKB, AR, CCND1, ERBB2, CDK2, MDM2, E2F1, BCL2, AKT1, MAPK1, CTNNB1, RAF1, TP53, MAPK3 |
| 7 | hsa05145 | Toxoplasmosis | XIAP, TNF, RELA, CASP9, IKBKB, MAPK8, CASP8, CASP3, ALOX5, AKT1, MAPK1, LDLR, MAPK3, NOS2, CHUK, STAT1, BAD, IL10RA, STAT3, MAPK14, MAPK10, NFKBIA, CD40LG, IFNG, BCL2, BCL2L1 |
| 8 | hsa05160 | Hepatitis C | GSK3B, CDKN1A, CXCL8, TNF, RELA, EGFR, IKBKB, MAPK8, RXRA, AKT1, MAPK1, LDLR, MAPK3, CHUK, EGF, STAT1, BAD, STAT3, MAPK14, MAPK10, NFKBIA, CLDN4, IRF1, RAF1, PPARA, TP53 |
| 9 | hsa05210 | Colorectal cancer | GSK3B, JUN, BAD, FOS, MAPK10, CASP9, MAPK8, CCND1, MYC, CASP3, BCL2, BAX, BIRC5, AKT1, MAPK1, CTNNB1, RAF1, TP53, MAPK3 |
| 10 | hsa05205 | Proteoglycans in cancer | CDKN1A, ELK1, HIF1A, TNF, EGFR, ERBB3, CCND1, PLAU, MYC, CASP3, ERBB2, KDR, AKT1, MAPK1, MAPK3, PRKCB, MMP2, CAV1, STAT3, IGF2, PRKCA, MAPK14, MMP9, ESR1, VEGFA, MDM2, CTNNB1, RAF1, MET, TP53 |
| 11 | hsa05223 | Non-small cell lung cancer | RB1, PRKCB, BAD, EGF, PRKCA, EGFR, CASP9, RXRA, CCND1, CDK4, ERBB2, E2F1, AKT1, MAPK1, RAF1, TP53, MAPK3 |
| 12 | hsa05220 | Chronic myeloid leukemia | RB1, CDKN1A, CHUK, BAD, RELA, NFKBIA, IKBKB, CCND1, CDK4, MYC, MDM2, E2F1, AKT1, MAPK1, RAF1, TP53, BCL2L1, MAPK3 |
| 13 | hsa04066 | HIF-1 signaling pathway | CDKN1A, NOS2, PRKCB, NOS3, EGF, STAT3, SERPINE1, PRKCA, HIF1A, EGFR, RELA, HK2, VEGFA, IFNG, ERBB2, BCL2, AKT1, HMOX1, MAPK1, MAPK3 |
| 14 | hsa05222 | Small cell lung cancer | RB1, NOS2, CHUK, XIAP, PTGS2, RELA, CASP9, NFKBIA, IKBKB, RXRA, CCND1, CDK4, MYC, CDK2, E2F1, BCL2, AKT1, TP53, BCL2L1 |
| 15 | hsa05142 | Chagas disease (American trypanosomiasis) | JUN, CXCL8, NOS2, CHUK, SERPINE1, FOS, MAPK14, TNF, RELA, MAPK10, NFKBIA, IKBKB, MAPK8, CASP8, IFNG, IL1B, CCL2, AKT1, MAPK1, MAPK3 |
| 16 | hsa05164 | Influenza A | GSK3B, PRSS1, CXCL8, TNF, RELA, ICAM1, CASP9, IKBKB, MAPK8, CCL2, AKT1, MAPK1, MAPK3, JUN, PRKCB, STAT1, PRKCA, MAPK14, MAPK10, NFKBIA, IL1A, CXCL10, IFNG, IL1B, RAF1 |
| 17 | hsa05140 | Leishmaniasis | JUN, NOS2, NCF1, STAT1, FOS, PTGS2, MAPK14, ELK1, TNF, RELA, IL4, NFKBIA, IL1A, IFNG, IL1B, MAPK1, MAPK3 |
| 18 | hsa04620 | Toll-like receptor signaling pathway | JUN, CXCL8, CHUK, STAT1, FOS, MAPK14, TNF, RELA, MAPK10, NFKBIA, IKBKB, CXCL10, CXCL11, MAPK8, CASP8, IL1B, SPP1, AKT1, MAPK1, MAPK3 |
| 19 | hsa05213 | Endometrial cancer | GSK3B, BAD, EGF, ELK1, EGFR, CASP9, CCND1, MYC, ERBB2, AKT1, MAPK1, CTNNB1, RAF1, TP53, MAPK3 |
| 20 | hsa04210 | Apoptosis | CHUK, BAD, XIAP, TNF, RELA, CASP9, NFKBIA, IKBKB, CASP7, CASP8, CASP3, BCL2, BAX, AKT1, TP53, BCL2L1 |

**Table S5 The value of the network topology parameters of the active ingredient**

| NO. | Mol ID | Degree | NO. | Mol ID | Degree | NO. | Mol ID | Degree |
| --- | --- | --- | --- | --- | --- | --- | --- | --- |
| 1 | MOL000098 | 124 | 37 | MOL000417 | 17 | 73 | MOL004805 | 10 |
| 2 | MOL000006 | 48 | 38 | MOL004857 | 16 | 74 | MOL002311 | 10 |
| 3 | MOL000422 | 47 | 39 | MOL004849 | 16 | 75 | MOL004945 | 9 |
| 4 | MOL003896 | 31 | 40 | MOL004820 | 16 | 76 | MOL004941 | 9 |
| 5 | MOL004328 | 30 | 41 | MOL005012 | 15 | 77 | MOL004913 | 9 |
| 6 | MOL000497 | 26 | 42 | MOL004915 | 15 | 78 | MOL004898 | 9 |
| 7 | MOL000392 | 26 | 43 | MOL004911 | 15 | 79 | MOL002844 | 9 |
| 8 | MOL000354 | 26 | 44 | MOL004907 | 15 | 80 | MOL004914 | 8 |
| 9 | MOL002565 | 25 | 45 | MOL004885 | 15 | 81 | MOL004829 | 8 |
| 10 | MOL000358 | 25 | 46 | MOL004864 | 15 | 82 | MOL004806 | 8 |
| 11 | MOL000449 | 24 | 47 | MOL004841 | 15 | 83 | MOL001792 | 8 |
| 12 | MOL000500 | 23 | 48 | MOL004808 | 15 | 84 | MOL005001 | 7 |
| 13 | MOL004891 | 23 | 49 | MOL003656 | 15 | 85 | MOL004948 | 7 |
| 14 | MOL007213 | 23 | 50 | MOL005020 | 14 | 86 | MOL004882 | 7 |
| 15 | MOL012921 | 23 | 51 | MOL004961 | 14 | 87 | MOL000492 | 7 |
| 16 | MOL004978 | 22 | 52 | MOL004856 | 14 | 88 | MOL002322 | 6 |
| 17 | MOL004957 | 22 | 53 | MOL004810 | 14 | 89 | MOL004989 | 6 |
| 18 | MOL004835 | 22 | 54 | MOL005008 | 13 | 90 | MOL004910 | 6 |
| 19 | MOL000627 | 22 | 55 | MOL005000 | 13 | 91 | MOL000096 | 6 |
| 20 | MOL005003 | 21 | 56 | MOL004990 | 13 | 92 | MOL012976 | 6 |
| 21 | MOL004974 | 21 | 57 | MOL004884 | 13 | 93 | MOL005018 | 5 |
| 22 | MOL002773 | 21 | 58 | MOL004883 | 13 | 94 | MOL004993 | 5 |
| 23 | MOL004991 | 20 | 59 | MOL004827 | 13 | 95 | MOL004988 | 5 |
| 24 | MOL004966 | 20 | 60 | MOL004904 | 12 | 96 | MOL004903 | 5 |
| 25 | MOL004959 | 20 | 61 | MOL004879 | 12 | 97 | MOL004838 | 5 |
| 26 | MOL004908 | 19 | 62 | MOL004866 | 12 | 98 | MOL004935 | 4 |
| 27 | MOL004833 | 19 | 63 | MOL004863 | 12 | 99 | MOL000359 | 4 |
| 28 | MOL000787 | 19 | 64 | MOL004848 | 12 | 100 | MOL001755 | 3 |
| 29 | MOL005007 | 18 | 65 | MOL001484 | 12 | 101 | MOL004924 | 3 |
| 30 | MOL004912 | 18 | 66 | MOL001454 | 12 | 102 | MOL000211 | 3 |
| 31 | MOL004828 | 18 | 67 | MOL005017 | 11 | 103 | MOL012992 | 3 |
| 32 | MOL004811 | 18 | 68 | MOL004814 | 11 | 104 | MOL002045 | 2 |
| 33 | MOL001522 | 18 | 69 | MOL000239 | 11 | 105 | MOL004350 | 2 |
| 34 | MOL005016 | 17 | 70 | MOL004980 | 10 | 106 | MOL012986 | 2 |
| 35 | MOL004824 | 17 | 71 | MOL004949 | 10 | 107 | MOL012946 | 2 |
| 36 | MOL004815 | 17 | 72 | MOL004855 | 10 |  |  |  |
